# MyC Factor Analogue CO5 Promotes the Growth of *Lotus japonicus* and Enhances Stress Resistance by Activating the Expression of Relevant Genes

**DOI:** 10.3390/jof10070458

**Published:** 2024-06-28

**Authors:** Xinhao Luo, Jiaqing Jiang, Jing Zhou, Jin Chen, Beijiu Cheng, Xiaoyu Li

**Affiliations:** 1 Schools of Life Sciences, Anhui Agricultural University, Hefei 230036, China; lxh1996@stu.ahau.edu.cn (X.L.); 19966101516@163.com (J.J.); 22710080@stu.ahau.edu.cn (J.Z.); chenjin131721@ahau.edu.cn (J.C.); 2 National Engineering Laboratory of Crop Stress Resistance Breeding, Anhui Agricultural University, Hefei 230036, China; 3 College of Agronomy, Anhui Agricultural University, Hefei 230036, China

**Keywords:** arbuscular mycorrhizal fungi, chitin oligomer 5, *Lotus japonicus*, hydroponic system

## Abstract

The symbiotic relationship between arbuscular mycorrhizal fungi (AMF) and plants is well known for its benefits in enhancing plant growth and stress resistance. Research on whether key components of the AMF colonization process, such as MyC factors, can be directly utilized to activate plant symbiotic pathways and key functional gene expression is still lacking. In this paper, we found that, using a hydroponics system with *Lotus japonicus*, MyC factor analogue chitin oligomer 5 (CO5) had a more pronounced growth-promoting effect compared to symbiosis with AMF at the optimal concentration. Additionally, CO5 significantly enhanced the resistance of *Lotus japonicus* to various environmental stresses. The addition of CO5 activated symbiosis, nutrient absorption, and stress-related signaling pathways, like AMF symbiosis, and CO5 also activated a higher and more extensive gene expression profile compared to AMF colonization. Overall, the study demonstrated that the addition of MyC factor analogue CO5, by activating relevant pathways, had a superior effect on promoting plant growth and enhancing stress resistance compared to colonization by AMF. These findings suggest that utilizing MyC factor analogues like CO5 could be a promising alternative to traditional AMF colonization methods in enhancing plant growth and stress tolerance in agriculture.

## 1. Introduction

The symbiotic relationship between plants and arbuscular mycorrhizal fungi is a common and beneficial interaction that exists in more than two-thirds of terrestrial plant species [1]. AMF obtain essential carbohydrates from their host plants and, in exchange, provide the plants with mineral nutrients and water absorbed from the soil [2]. Due to the lack of strict host specificity, AMF can establish connections between the root systems of different plants. Through the extraradical hyphal network, they facilitate the redistribution of resources among various plants, thereby maintaining the stability of plant community structure and function [3]. At the ecosystem level, AMF play a significant role in nutrient cycling and regulate the ecosystem’s response to environmental fluctuations, underscoring their irreplaceable ecological significance [4,5].

This symbiosis plays a crucial role in enhancing plant growth and stress tolerance. The expanded mycelium network of AMF is a key factor in enhancing the stress tolerance of host plants while also aiding in the absorption of water and nutrients from the soil [6]. Once a symbiotic relationship is established, plants can rely on AMF to enhance phosphorus uptake through phosphate transporters [7]. Additionally, increasing evidence suggests that AMF can absorb both NO_3_^−^ and NH_4_^+^, thereby helping plants acquire the essential nutrient nitrogen for growth and development [8,9]. Furthermore, numerous studies have shown that under abiotic stress, the expression of stress-related genes in host plants is influenced by AMF through different response mechanisms, such as those for drought, heavy metals, and high salinity [10,11,12].

The establishment of AMF symbiosis involves a molecular dialogue between the plant and fungal partners. Our understanding of this molecular interaction has been greatly informed by studies on rhizobium–legume symbiosis [13]. It is believed that the symbiosis involving fungal associations predates bacterial symbiosis, leading to the hypothesis that rhizobia utilize ancient mycorrhizal signaling pathways by mimicking signals used by symbiotic fungi [14]. In this context, it has been proposed that AMF release signaling molecules known as mycorrhiza (MyC) factors that are essential for recognition by plant partners. MyC factors are a mixture of lipochitooligosaccharides (LCOs) and chitin oligomers (COs). AM fungi produce diffusible symbiotic signals containing a combination of sulphated and non-sulphated simple LCOs [15]. COs, like in colonization by arbuscular mycorrhizal fungi, can trigger calcium spikes in the epidermal cells of plant roots and serve as a well-characterized elicitor of plant responses. They can activate the plant’s innate immune response by inducing the expression of genes associated with biotic stress, responses to phytopathogenic fungi [16]. Studies have shown that COs exhibit higher activity compared to both sulfated and non-sulfated MyC LCOs in inducing AM-dependent calcium spiking. This calcium spiking response is dependent on genes within the common SYM signaling pathway, particularly DMI1 and DMI2 [17]. This molecular dialogue between plant and fungal partners, mediated by MyC factors and COs, plays a crucial role in the establishment and functioning of AMF symbiosis.

In practical production applications, the establishment of a symbiotic relationship requires the extensive propagation of fungal spores. Additionally, the intricate conditions for spore propagation contribute to increased costs in preparing durable AM microbial fertilizer products. Therefore, it is crucial to investigate whether MyC factor analogues demonstrate symbiotic and growth-promoting effects, as well as stress resistance, by activating gene expressions associated with plants’ symbiotic pathways. Utilizing MyC factor analogues as regulators for plant growth in AMF symbiosis shows significant potential for enhancing plant growth and stress resistance in agricultural production.

To achieve this goal, we selected a representative chitin oligomer, CO5, as the material for investigating its potential growth-promoting effects on *Lotus japonicus*, like symbiosis with AMF. This investigation seeks to uncover the influence of externally applied MyC factor analogues on the growth, stress resistance, and signaling pathways of *Lotus japonicus* roots. By doing so, we aim to establish both experimental and theoretical frameworks for the potential application of MyC factor analogues as symbiotic regulators.

## 2. Materials and Methods

### 2.1. The Establishment of a Hydroponic System

Our hydroponic system was built using a 10 mL pipette tip box and customized pipettes. The growth medium consisted of high-temperature-resistant aluminum silicate asbestos (Figure S1). Subsequently, the germinative seeds were cultivated in Hoagland modified nutrient salts (−P) (Coolaber, Beijing, China) for 60 days in a growth chamber with a diurnal cycle of 16 h of light and 8 h of darkness, at temperatures of 26 °C during the day and 22 °C at night and a relative humidity of 70%.

### 2.2. Arbuscular Mycorrhizal Fungi and Mycorrhiza Factor Analogues

In this study, *Rhizophagus intraradices* associated with the plant were obtained from Sun Yat-sen University and extensively propagated for use. Chitin oligomer 5 (chitooligosaccharides, CO5, Purity ≥ 97.0%) was purchased from Yuanye BioTechnology Co., Ltd. (Shanghai, China). When applying AMF spores individually, 50 spores per plant were used (Figure S2); when applying CO5 individually, the optimal concentration chosen was 10 nM (Figure S3).

### 2.3. Plant Experiment Design

The host plant, *Lotus japonicus* ecotype MG20, was maintained in our laboratory for experimental purposes. *Lotus japonicus* seeds were sterilized using a 12% (*v*/*v*) KAO bleach solution (KAO Industrial Co. Ltd., Hong Kong, China) and 75% (*v*/*v*) alcohol, followed by rinsing with distilled water. After sterilization, the *Lotus japonicus* seeds were placed on a 1.2% agar medium and incubated in the dark at 4 °C for 12 h for vernalization. Subsequently, the seeds were transferred to a growth chamber set at 28 °C for further cultivation [18].

To evaluate the impact of externally applied fungal factor analogues on *Lotus japonicus* growth, different experimental groups were established, including a control group (CK), a group with AMF spores, and groups treated with individual fungal factor analogues (CO5). AMF spores were inoculated at the top of the rockwool pieces using a pipettor, followed by transplantation. CO5 was directly added to the hydroponic nutrient solution.

To assess the impact of externally applied fungal factor analogues on the stress resistance of *Lotus japonicus*, a control group and experimental groups with externally applied optimal fungal factor analogues were established for positive and negative control experiments. Initially, *Lotus japonicus* plants were cultivated in a hydroponic system with a standard Hoagland modified nutrient salts solution for 30 days. Then, treatments were applied according to the specific stress conditions. For high-temperature or low-temperature stress, the hydroponic system and plants were transferred to a 42 °C incubator or a 4 °C refrigerator one day before sampling. For low-nitrogen or low-phosphorus stress, the nutrient solution was replaced with Hoagland modified nutrient salts solution (−N) or Hoagland modified nutrient salts solution (−P) 7 days prior to sampling. Similarly, for high-salinity stress, the nutrient solution was replaced with 150 mM sodium chloride, and for drought stress, 200 g of PEG 6000 was added to the nutrient solution per system. All types of Hoagland nutrient solutions were purchased from Coolaber (Beijing, China). PEG-6000 was purchased from Solarbio (Beijing, China).

### 2.4. The Determination of Some Physiological and Biochemical Indexes

After sampling, the plants were separated into their shoot and root components and individually weighed. The cumulative fresh weight was recorded as the total biomass, and the ratio of root weight to shoot weight determined the root-to-shoot ratio.

The levels of soluble sugar, peroxidase (POD), malondialdehyde (MDA) and proline (PRO) were analyzed using ELISA assay kits following the instructions provided by Nanjing Jiancheng Bioengineering Institute (Nanjing, China). All assay kits were purchased from the institute for accurate and standardized measurements.

### 2.5. Trypan Blue Staining

Trypan blue staining of mycorrhizal roots from *Lotus japonicus* was executed following established protocols [19]. Root segments of *Lotus japonicus* were first fixed in FAA (formalin acetic acid alcohol) for 4 h. Subsequently, the root segments were subjected to high-temperature incubating with 10% KOH, acidified, and made transparent. Finally, the root segments were stained with a 0.05% trypan blue solution. 

The AM fungal structures within root segments were then visualized using DM5000B microscope from Leica (Wetzlar, Germany). The determination of the root colonization rate was carried out following a methodology previously published [18]. 

### 2.6. RNA Sequencing and Differentially Expressed Gene Analysis

The sequencing of libraries was carried out on an Illumina HiSeq X Ten platform, producing 150 bp paired end reads in fastq format. The raw data underwent initial processing using Trimmomatic to eliminate low-quality reads and obtain clean reads [20]. Approximately, a certain number of clean reads were retained for each sample for subsequent analyses.

FPKM values were calculated for each gene using Cufflinks, and read counts were obtained using HTSeq-count [21,22]. The DESeq (2012) R package was employed for conducting the differential expression analysis, with a significance threshold set at a *p* value < 0.05 and a fold change > 2 or <0.5 for significantly differentially expressed genes (DEGs). Hierarchical cluster analysis was performed to visualize the expression pattern of genes across different groups and samples. Furthermore, GO enrichment and KEGG pathway enrichment analyses of DEGs were performed using R based on the hypergeometric distribution [23]. 

Total RNA was isolated using the phenol–chloroform extraction method, followed by the synthesis of first-strand cDNAs from DNaseI-treated total RNA using reverse transcriptase (TransGen, Beijing, China) and oligo-dT primers. To normalize gene expression, internal controls including the *ubiquitin* gene and *ATPase* genes were employed. Subsequently, qPCR was performed on an Applied Biosystems 7300 system with ChamQ Universal SYBR qPCR Master Mix (Vazyme, Nanjing, China) following the manufacturer’s protocol. The relative gene expression levels were determined using the 10^(ΔΔCt/3) method. The qPCR assays were conducted with three biological replicates.

### 2.7. Statistical Analysis 

The results are presented as the mean followed by the standard error. The data were analyzed at a significance level of 0.05 using the Student’s two-tailed *t*-test, one-way ANOVA, or two-way ANOVA, as appropriate. Post hoc comparisons between three or more groups were conducted using Tukey’s test to determine significant differences.

## 3. Results

### 3.1. Analysis of Growth-Promoting Effects of CO5 on Lotus japonicus

**Figure 1:**
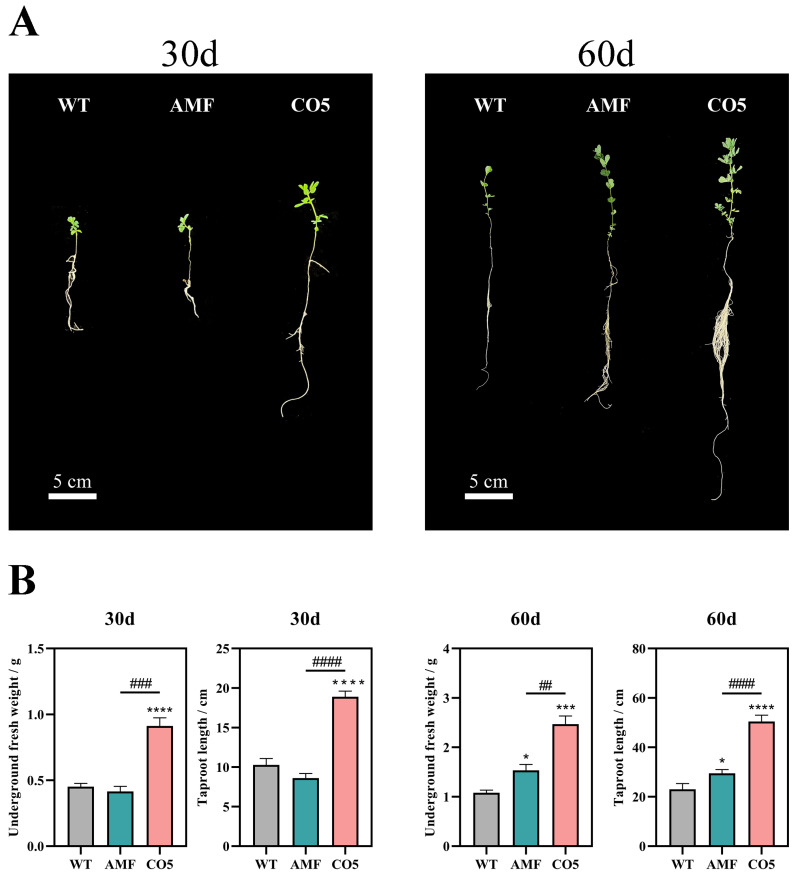
Analysis of the growth-promoting effects of CO5 on *Lotus japonicus*. (**A**) The effect of MyC factor analogue CO5 on *Lotus japonicus*. (**B**) Underground fresh weight and taproot length of *Lotus japonicus* at 30 and 60 days. * *p* < 0.05, *** *p* < 0.001 **** *p* < 0.0001; ## *p* < 0.01, ### *p* < 0.001, #### *p* < 0.0001 (n = 3, data are the means ± SEM).

In the hydroponic system of *Lotus japonicus*, the application of exogenous CO5 was implemented, and samples were collected for observation at cultivation durations of 30 and 60 days. The addition of CO5 at these two distinct time points resulted in a significant enhancement of *Lotus japonicus* growth. Compared to the untreated group and the group inoculated with AMF, the CO5-treated plants displayed more robust growth, characterized by increased foliage aboveground and a more extensive and densely populated root system underground (Figure 1A). Subsequently, an analysis of taproot length and underground fresh weight of *Lotus japonicus* revealed that the CO5-treated group exhibited significant superiority over the untreated group, while also demonstrating a pronounced advantage over the AMF-inoculated group (Figure 1B). In comparison to symbiosis with AMF, the addition of CO5 exerts a more prominent growth-promoting effect on *Lotus japonicus*. Interestingly, at the 30-day time point, the growth status of the AMF-inoculated plants was not superior to that of the untreated group. This lack of superiority was evident in both taproot length and underground fresh weight, where no significant difference was observed between the AMF group and the untreated group.

### 3.2. Analysis of Stress Resistance Enhancement Effects of CO5 on Lotus japonicus

**Figure 2:**
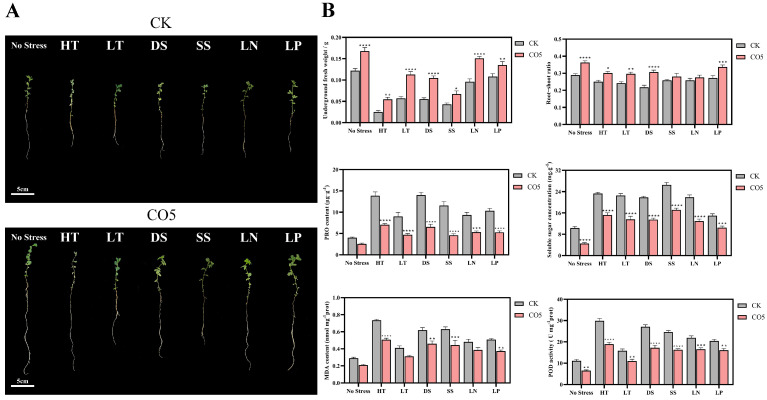
Analysis of the stress resistance enhancement effects of CO5 on *Lotus japonicus*. (**A**) The effect of CO5 on different stresses of *Lotus japonicus*. (**B**) Underground fresh weight, root–shoot ratio, and the physiological indexes of different stresses of CO5 on *Lotus japonicus*. * *p* < 0.05, ** *p* < 0.01, *** *p* < 0.001, **** *p* < 0.0001 (n = 3, data are the means ± SEM).

To investigate the potential of CO5 in promoting the growth of *Lotus japonicus* under stress conditions, seven common stress treatments were applied, including high-temperature stress (HT), low-temperature stress (LT), drought stress (DS), high-salt stress (SS), low-nitrogen stress (LN), and low-phosphorus stress (LP). The results showed that the addition of CO5 effectively alleviated the growth inhibition of *Lotus japonicus* under these stress conditions. The relief was evident in the increased leaf number and root length observed in the CO5-treated plants (Figure 2A). Additionally, the underground fresh weight and root–shoot ratio of the CO5-treated group exhibited significant increases compared to the untreated group (Figure 2B). Furthermore, physiological indicators reflective of stress severity, including MDA, POD, PRO, and soluble sugars, were measured. The addition of CO5 was found to significantly reduce the levels of all four stress indicators (Figure 2B). Overall, the findings suggest that CO5 has the potential to enhance the stress resistance of *Lotus japonicus* under various stress conditions. 

### 3.3. Dynamic Changes and Comparative Analysis of DEGs

To evaluate the impact of the MyC factor analogue CO5 on the expression pathway of *Lotus japonicus*, RNA-seq sequencing was conducted on three distinct treatments: the untreated wild-type (WT) group, the AMF group treated with R.i spores, and the experimental group treated with the MyC factor analogue CO5. In this study, q-value < 0.05 and fold change > 2 were used as thresholds to statistically analyze the transcriptome data. 

**Figure 3:**
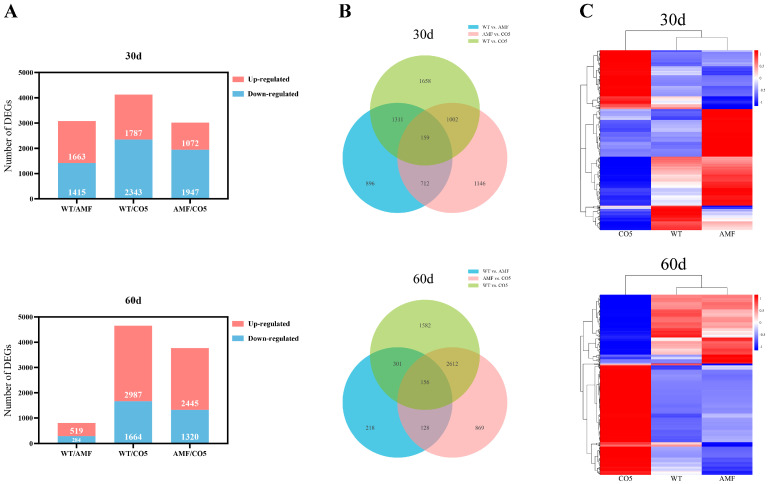
Dynamic changes and comparative analysis of DEGs. (**A**) Number of transcriptome expression changes in different comparison groups at different periods. (**B**) Number of DEGs between WT, AMF, and CO5. (**C**) Heat map of signaling pathways and related gene expression.

The number of DEGs between two developmental stages was as follows: at 30 days, 3078 between WT and AMF (1663 up- and 1415 down-regulated in AMF), 4130 between WT and CO5 (1787 up- and 2343 down-regulated in CO5), and 3019 between AMF and CO5 (1072 up- and 1947 down-regulated in CO5); at 60 days, 803 between WT and AMF (519 up- and 284 down-regulated in AMF), 4651 between WT and CO5 (2987 up- and 1664 down-regulated in CO5), and 3765 between AMF and CO5 (2445 up- and 1320 down regulated in CO5) (Figure 3A). In comparison to AMF, CO5 demonstrated a greater number of DEGs between the WT group at both 30 and 60 days. This conclusion is further supported by the Venn diagram. Additionally, the shared DEGs among the three comparisons (159 at 30 days and 156 at 60 days) should be a focal point of our subsequent investigations (Figure 3B). From the expression pattern depicted in the heatmap, the WT and AMF groups clustered together, while CO5 formed a distinct cluster on its own. Especially at 60 days, the expression pattern of DEGs in CO5 showed a distinct contrast with the other two groups. And it was noteworthy that, compared to WT, the cluster of genes exhibiting high expression in CO5 tended to have lower expression in AMF. Conversely, genes showing high expression in AMF often experienced decreased expression in CO5 (Figure 3C). These categories of DEGs deserved particular attention in our subsequent investigations.

### 3.4. Gene Ontology Classification and Pathway Enrichment Analysis of DEGs

**Figure 4:**
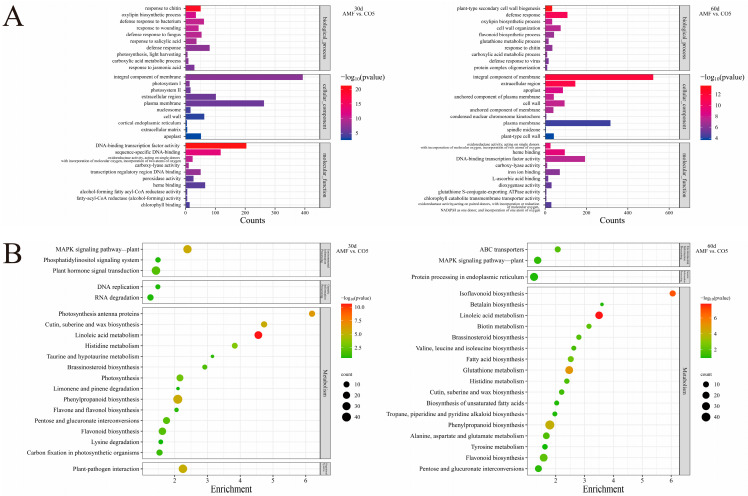
Gene ontology classification and Pathway Enrichment Analysis of DEGs. (**A**) The most enriched GO term of AMF and CO5. (**B**) The most enriched KEGG term of AMF and CO5.

To gain a deeper understanding of the biological functions of genes within these modules, comprehensive GO and KEGG enrichment analyses were conducted, with a focus on the comparison between AMF and the MyC factor analogue CO5 (Figure 4). In the GO analysis, the distribution of DEGs was examined in the Biological Process, Cellular Component, and Molecular Function categories. The top three enriched pathways were integral components of membrane, plasma membrane, and DNA-binding transcription factor activity (Figure 4A). As the time progressed from 30 to 60 days, there was a further increase in the enrichment of the integral component of the membrane, while the enrichment in the other two pathways decreased. In the KEGG analysis, the DEGs of AMF vs. CO5 were predominantly enriched in the metabolism pathway. At 30 days, photosynthetic antenna proteins, cutin, suberine, and wax biosynthesis, and linoleic acid metabolism exhibited significant enrichment and a higher number of enriched genes. In contrast, at 60 days, the enriched pathways were primarily Isoflavonoid biosynthesis and linoleic acid metabolism (Figure 4B). These findings suggest that the presence of CO5 has a significant influence on various metabolic processes and biological pathways within the plant. This lays a foundational basis for further investigation into the effects of CO5 on the molecular mechanisms and physiological processes of *Lotus japonicus*.

### 3.5. CO5 Activates the Symbiotic Pathway, Nitrogen and Phosphorus Absorption, and Disease-Resistant and Stress-Related Gene Expression

**Figure 5:**
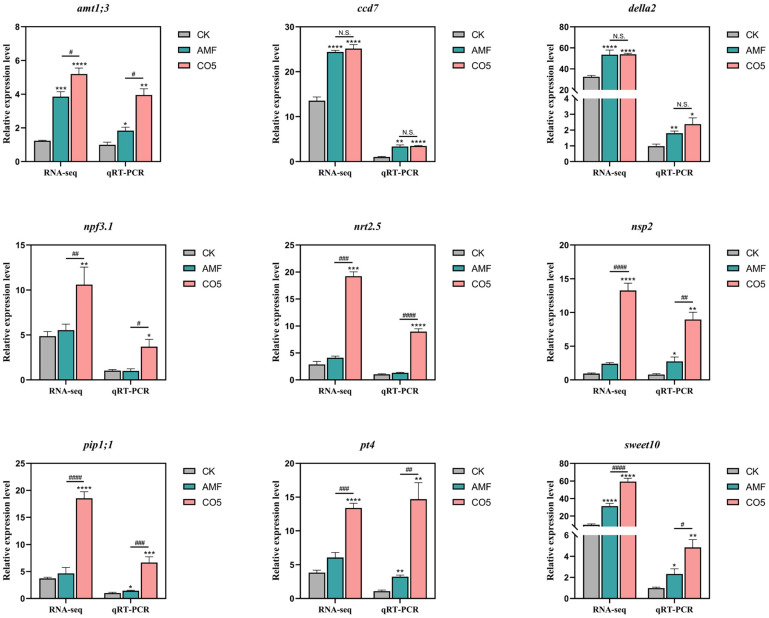
CO5 activates the symbiotic pathway, nitrogen and phosphorus absorption, and disease-resistant and stress-related gene expression. * *p* < 0.05, ** *p* < 0.01, *** *p* < 0.001, **** *p* < 0.0001; # *p* < 0.05, ## *p* < 0.01, ### *p* < 0.001, #### *p* < 0.0001 (n = 3, data are the means ± SEM).

By further analyzing the genes involved in the symbiotic signaling pathway within the GO and KEGG pathways, several crucial genes were identified as involved in various stages, such as Nsp in rhizodermal penetration, Della in trunk formation, Ram and Rad in fine branch formation, Myb in maintenance degeneration, etc. These genes play a vital role in the establishment and degradation of arbuscular morphology (Table 1). At 60 days, the expression levels of Ccd7, Nsp2, and Della2 in the CO5 group were significantly higher compared to the CK group and show no significance with the AMF group, indicating that the analogue CO5 can substitute for symbiosis with AM fungi and activate the symbiotic pathway. Additionally, genes related to nitrogen absorption and transporters, such as phosphate transport protein PT, AMT for nitrogen absorption NPF, and NRT transporters for NO^3−^ and oligopeptide transport, were identified. Notably, genes associated with disease and stress resistance, including PIP and SWEET, were also detected (Table 2). At 60 days, the expression levels of Amt1;3, Npf3.1, Nrt2.5, Pt4, Pip1;1, and Sweet10 in the CO5 group were significantly elevated compared to the WT group, and the increase was also significant compared to the AMF group. Taken together, these results suggested that the MyC factor analogue CO5 not only acts as a substitute for AMF symbiosis but also effectively alleviates plant diseases and stress-related issues, thereby promoting favorable plant growth (Figure 5).

## 4. Discussion

AMF are important symbiotic microorganisms in soil that form arbuscular mycorrhizal associations with host plants. Once a stable symbiotic relationship is established with plants, AMF engage in nutrient exchange through arbuscular branches and hyphae, benefiting both the fungi and the plants [24]. Studies have shown that AMF produce diffusible fungal signals known as MyC factors, which can be derived from AM hyphae, germinating spores, or mycorrhizal roots [25]. These MyC factors are primarily composed of lipochitooligosaccharides and short-chain chitin oligomers [15,17]. Previous research has indicated that MyC factors can induce intense Ca_2_^+^ spiking and the associated intensity [15,17,26], highlighting their potential role in signal transduction, cell communication, and environmental stimuli responses [27]. The main objective of this study was to investigate whether a representative MyC factor analogue CO5 could effectively replace the inoculation of AM fungi and achieve comparable or even superior effects compared to AMF symbiosis. This research aimed to explore the potential of CO5 in promoting plant growth and enhancing stress resistance, ultimately contributing to the development of innovative strategies for sustainable agriculture.

In the experiments, the addition of the MyC factor analogue CO5 could simulate the symbiotic effect of AM fungi with *Lotus japonicus*. And this simulation promotes lateral root branching, enhances primary root length, and increases underground fresh weight, thereby achieving a growth-promoting effect on *Lotus japonicus* (Figure 1). This is consistent with the work of Sonja Kosuta et al. in 2003, which facilitated lateral root branching to promote the growth of Medicago truncatula [28]. Low-molecular-weight forms of chitin, such as CO5, may have a role in nature as biostimulators of plant growth. Additionally, they are recognized as a direct source of carbon and nitrogen for biomass [16]. Furthermore, our experiments indicate that the addition of CO5 can assist *Lotus japonicus* in better adapting to adverse stress conditions (Figure 2). The stress resistance effects resulting from the symbiosis between arbuscular mycorrhizal fungi and plants have been extensively studied [29,30,31,32,33]. Our research demonstrates that the addition of the isolated MyC factor analogue CO5 also exhibits stress resistance effects. This is consistent with the findings of Devanshi Khokhani et al., who summarized that substances like chitin oligomers can serve as elicitors of plant immune responses, activating defense signaling pathways [34]. 

Through RNA-seq, we observed that, compared to colonization by AMF, the sole addition of CO5 resulted in a greater number of differentially expressed genes, involving variations in enrichment across various GO and KEGG pathways. The prominent pathway identified was the integral component of the membrane (GO:0016021) and linoleic acid metabolism (map00591) (Figure 4). Integral components of the membrane refer to membrane proteins that play a crucial role in cellular signal transduction, cellular recognition, and transport [35]. Linoleic acid functions in plant defense signaling and stress tolerance [36]. For instance, the Arabidopsis FAD2 omega-6 desaturase localizes to the endoplasmic reticulum and has been demonstrated to be crucial for salt tolerance during germination [37]. 

In the RAM1 gene, which is specifically required for AMF symbiosis [38], the gene expression in the AMF group is significantly higher than that in the CO5 group at 60 days. During the early stages of symbiosis, the main signal communication between AMF and host plants involves the mutual recognition of small molecular substances [39]. Upon the mycelium reaching the endodermis, an essential interaction for the subsequent symbiosis, namely the formation of arbuscular structures, involves the indispensable interaction with CYCLOPS, where DELLA plays a crucial role. Simultaneously, DELLA also plays a significant role in maintenance degeneration [40,41]. RAD17 is also a member of the GRAS family protein, and it has been reported to possess functions in meiosis [42], being required for responses to DNA damage, replication stress, and double-strand break (DSB) repair [43]. As a member of the same family as RAD1, RAD17 may also interact with RAM1. The establishment of AMF symbiotic relationships requires the involvement of plant root-derived strigolactones (SLs) [44]. And CCD7 encodes a key enzyme in the synthesis of SLs [45]. In the maintenance degeneration stage, apart from DELLA, MYB and NSP are also two crucial transcription factors. MYB, on one hand, could act as a regulator of some mycorrhizal-responsive genes in arbusculated cells, and on the other, it could be involved in the mechanisms that regulate root growth [27]. NSP1 and NSP2 coordinate fungal and plant processes associated with LCO production and perception, facilitating AMF symbiosis [46]. In the relative expression levels obtained from the RNA-seq, except for RAM1, the expression of other key genes in the CO5 group is either higher or not significantly different from that of the AMF group. It has been demonstrated that at the symbiotic level, the addition of CO5 can, to some extent, substitute the role of AMF.

Ammonium is a preferred source of nitrogen for plants, and AMTs play an essential role in NH_4_^+^ uptake [47]. Many members of the nitrate transporter 1/peptide transporter family (NPF), including NPF and NRT, are also involved in the translocation of nitrogenous compounds such as nitrate, amino acids, peptides, and plant hormones [48,49]. 

A transcriptome analysis revealed that the down-regulation of NRT3.1 nitrate transporter genes plays a significant role in the rhizobium symbiosis-induced tolerance of Medicago truncatula to arsenate [50]. After the formation of arbuscular structures, there is a substantial exchange of nutrients between AMF and host plants. For example, the phosphate transport protein PT, induced by AM fungi, begins to acquire phosphate from the membranes surrounding the arbuscules [51,52]. SWEET transporters represent a unique class of sugar transporters that play crucial roles in various developmental and physiological processes in plants [53]. Simultaneously, they are closely associated with pest resistance [54]. Plasma membrane intrinsic proteins (PIPs) in root cortical cells are crucial for water uptake in plants and are believed to be directly involved in cell growth [55]. Aquaporins, to which PIPs belong, are membrane channels that play an essential role in maintaining cellular water and osmotic homeostasis in plants under both normal and water-deficit conditions [56]. In these genes related to the regulation of nitrogen and phosphorus transport as well as stress resistance, the gene expression levels in the CO5 group are significantly higher than in the AMF group at 60 days. This demonstrates that the addition of CO5 surpasses AMF in promoting growth and stress resistance levels. 

However, the specific mechanisms by which isolated CO5 can substitute for the symbiotic effects of AMF still require further investigation. As we described before, in previous studies, MyC factors, including CO5, have been identified as the primary signaling molecules activating symbiotic responses, such as calcium oscillations, during the symbiosis between plants and AMF [57]. Although there are no related reports, we believe that CO5 plays a crucial role in symbiosis and can function independently of it. For example, strigolactones, which are other plant signaling molecules of AMF, can also function as plant hormones, independently promoting plant growth outside of the symbiotic process [58,59]. 

## 5. Conclusions

In this study, we found that, compared to colonization by AMF, the addition of MyC factor analogue CO5, through the activation of relevant pathways, has a superior effect on promoting plant growth and enhancing stress resistance. It can regulate different stages of plant growth and development, promoting lateral root branching and activating signaling pathways related to nitrogen and phosphorus absorption, as well as plant stress resistance. In the future, it may be possible to produce seed coating agents containing MyC factor analogue CO5. Different crop seeds can be enveloped in CO5 at the optimal concentration. This seed coating, applied during the early stages of crop growth and development, could directly stimulate plants to activate symbiotic signaling pathways, thereby achieving effects comparable to those of AMF.

## Figures and Tables

**Table 1:** Expression of genes related to symbiotic pathway activated by analogue CO5.

Gene Symbol	Description
*NSP1*	Nitrile-specifier protein
*NSP2*	Protein Nodulation Signaling Pathway
*NIN3*	Neutral/alkaline invertase, chloroplastic
*RAM1*	GRAS family protein
*RAD17*	Cell cycle checkpoint protein
*RAD23B*	Ubiquitin receptor
*RAD1*	Cell cycle checkpoint protein
*RAD5A*	DNA repair protein
*RAD52*-*2*	DNA repair RAD52-like protein 2, chloroplastic
*RAD5B*	DNA repair protein
*MYB1*	Transcription factor
*MYB2*	
*MYB11*	
*MYB17*	
*RMS3*	Strigolactone esterase
*DELLA1*	DELLA protein 1
*DELLA2*	
*DAD1*	Dolichyl-diphosphooligosaccharide-protein glycosyltransferase subunit
*CCD7*	Carotenoid cleavage dioxygenase, chloroplastic
*CCD8*	

**Table 2:** Gene expression related to nitrogen and phosphorus uptake and resistance pathways activated by analogue CO5.

Related Signal Pathway	Gene Symbol	Description
Phosphorus absorption	*PHT1;1*	Inorganic phosphate transporter
	*PHT2*	
	*PTS1*	Pterocarpan synthase
	*PT1*	Low-affinity inorganic phosphate transporter
	*PHR2*	Blue-light photoreceptor
	*HAM1*	Histone acetyltransferase of the MYST family
	*SHR*	Protein SHORT-ROOT
	*PAP2*	Poly(A) RNA polymerase protein
	*SPS3*	Solanesyl diphosphate synthase, chloroplastic/mitochondrial
	*PHO1*	Alpha-1,4 glucan phosphorylase L isozyme, chloroplastic/amyloplastic
Nitrogen absorption	*NLP3*	Omega-amidase, chloroplastic
	*NLP7*	Omega-amidase, chloroplastic
	*NRT3.1*	High-affinity nitrate transporter
	*SPX4*	SPX domain-containing protein
	*CHL*	Chloroplastic lipocalin
	*ZFP1*	Zinc finger protein
	*ZFP3*	
	*NPF6.3*	Protein NRT1/PTR FAMILY
	*NPF8.3*	
	*NPF3.1*	
Disease resistance	*AMT1;3*	Ammonium transporter
	*AMT2*	
	*CWINV3*	Beta-fructofuranosidase, insoluble isoenzyme
	*FBL3*	Putative F-box/LRR-repeat protein
	*FBL8*	
	*SAG20*	Senescence-associated gene
	*SWEET5*	Protein SWEETIE
	*SWEET10*	
Stress resistance	*AQP1*	Probable aquaporin TIP-type
	*bHLH110*	Transcription factor
	*TIP1;3*	Aquaporin
	*HVA22*	Protein
	*P5CS*	Gamma-glutamyl phosphate reductase
	*LEA14*-*A*	Late embryogenesis abundant protein

## Data Availability

The data presented in this study are available in this article or Supplementary Material.

## Supplementary Materials

The following supporting information can be downloaded at: https://www.mdpi.com/article/10.3390/jof10070458/s1, Figure S1: The planting process of a hydroponic device; Figure S2: The effect of different spore number on the growth of *Lotus japonicus*; Figure S3: Root–shoot ratio of *Lotus japonicus* at different concentrations of CO5.
