# Supplementary material for: MyC Factor Analogue CO5 Promotes the Growth of Lotus japonicus and Enhances Stress Resistance by Activating the Expression of Relevant Genes
Source: J Fungi (Basel). 2024 Jun 28;10(7):458. doi: 10.3390/jof10070458 (PMC11278419; doi:10.3390/jof10070458)
Supplement: Supplementary file 1 [file jof-10-00458-s001.zip › jof-3028698-supplementary.pdf]

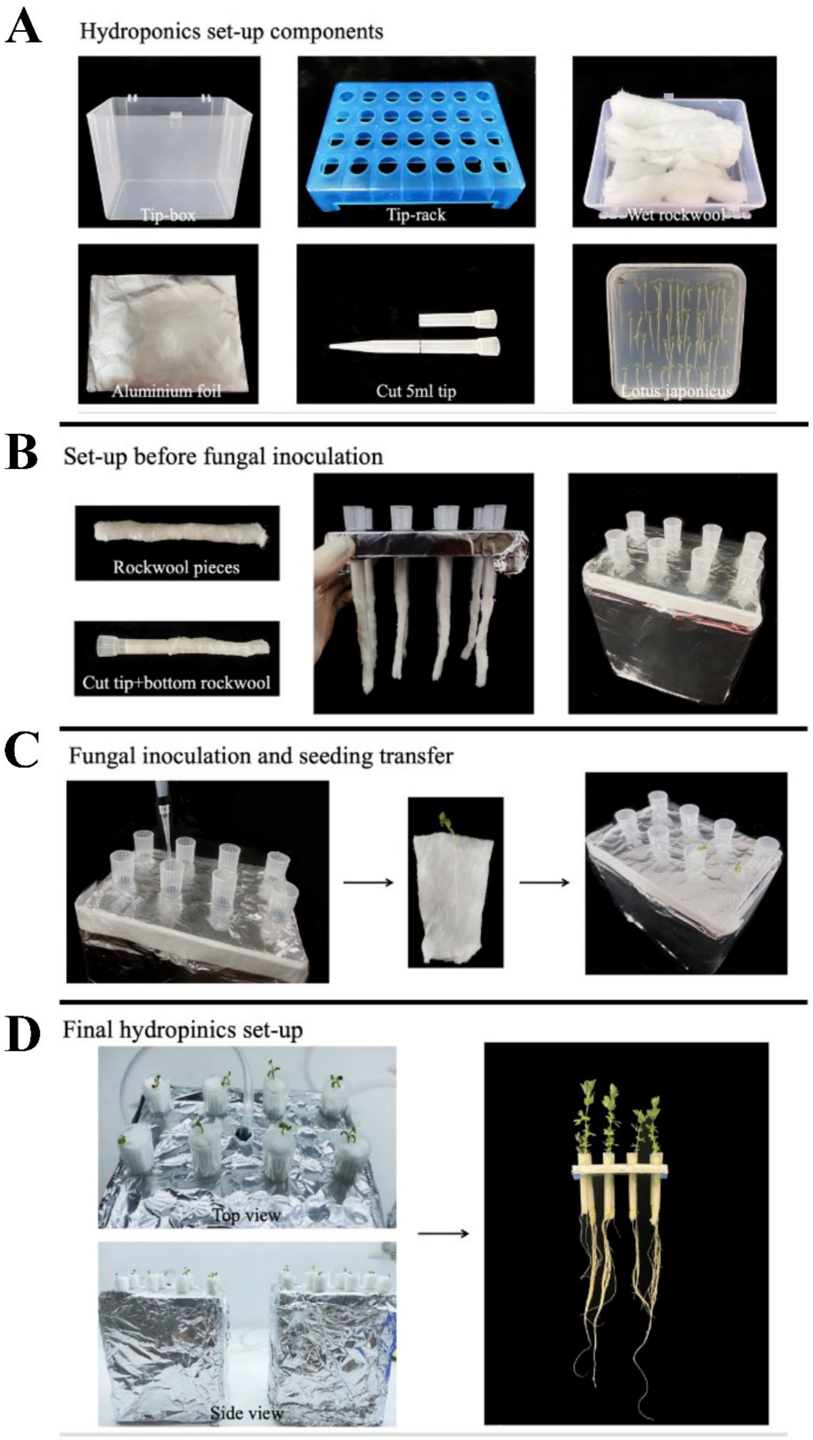

Figure S1. The planting process of a hydroponic device.

A. Hydroponics set-up components. B. Set-up before fungal inoculation. C. Fungal inoculation and seeding transfer. D. Final hydropinics set-up.

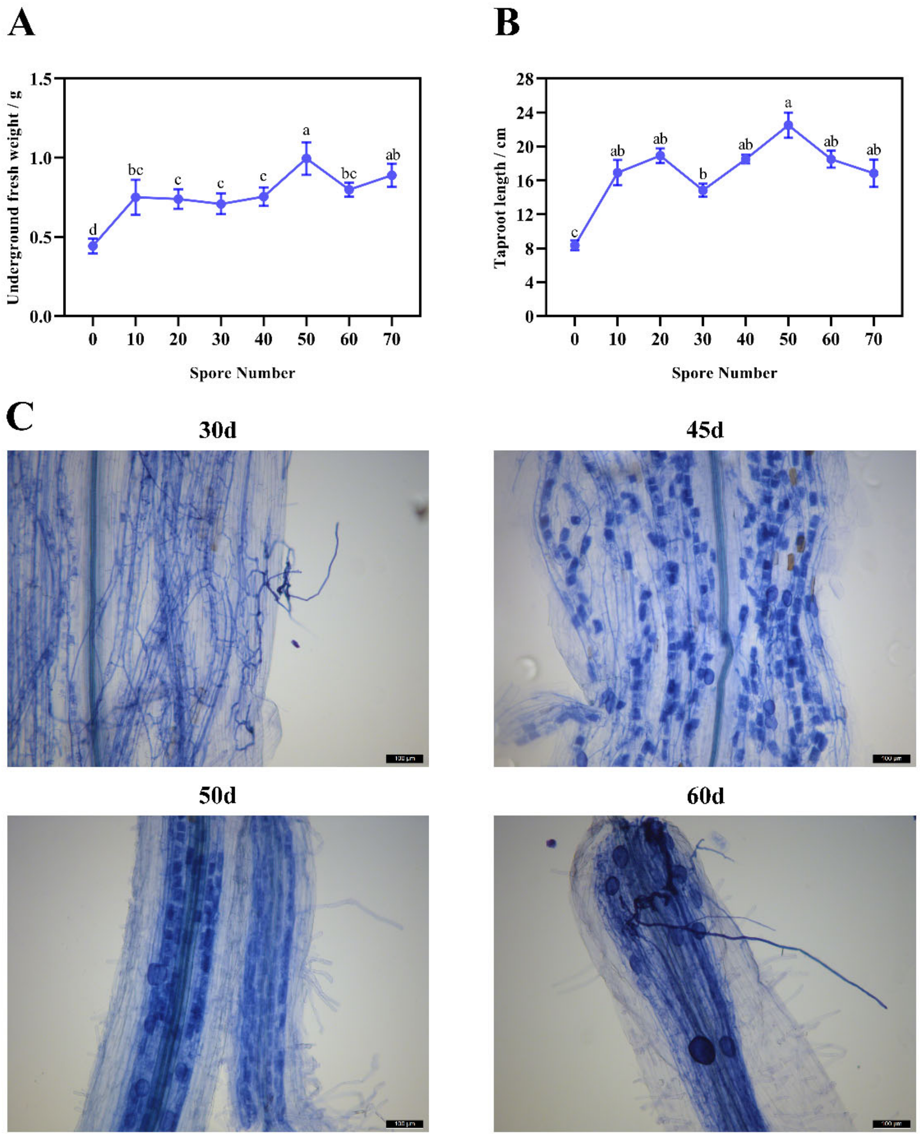

Figure S2. Effect of different spore number on the growth of *Lotus japonicus*

A. Underground fresh weigh of *Lotus japonicus*. B. Taproot length of *Lotus japonicus*. C. Fungal inoculation and seeding transfer. D. 30 days, 45 days, 50 days and 60 days mycorrhizal staining diagram. [a, b, c, d, e] Different lowercase letters in each column indicate significant differences among cultivars ($p < 0.05$), data are presented as the mean ± standard deviations of three replicates.

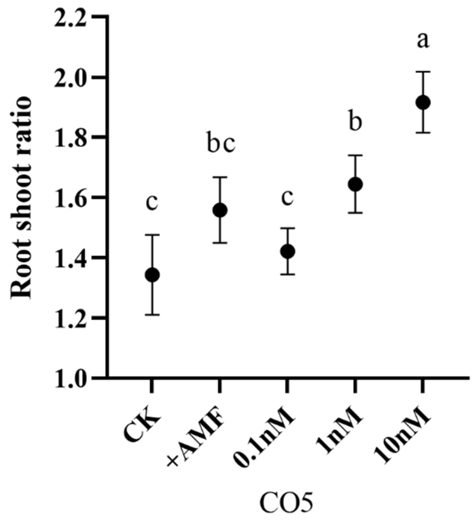

Figure S3. Root shoot ratio of *Lotus japonicus* at different concentrations of CO5.
